# Development and Validation of Computational Fluid Dynamics Model of Ladle Furnace with Electromagnetic Stirring System

**DOI:** 10.3390/ma17040960

**Published:** 2024-02-19

**Authors:** Monika Zielinska, Hongliang Yang, Lukasz Madej, Lukasz Malinowski

**Affiliations:** 1 Corporate Technology Center CTC, ABB Sp. z o. o., 31-038 Kraków, Poland; monika.zielinska@pl.abb.com (M.Z.); lukasz.malinowski@pl.abb.com (L.M.); 2 Department of Applied Computer Science and Modelling, AGH University of Krakow, 30-059 Kraków, Poland; 3 ABB AB/Metallurgy, SE-72159 Västerås, Sweden; hongliang.yang@se.abb.com

**Keywords:** metallurgy, computational fluid dynamics, electromagnetic stirring, ladle furnace, validation

## Abstract

Numerical methods are crucial to supporting the development of new technology in different industries, especially steelmaking, where many phenomena cannot be directly measured or observed under industrial conditions. As a result, further designing and optimizing steelmaking equipment and technology are not easy tasks. At the same time, numerical approaches enable modeling of various phenomena controlling material behavior and, thus, understanding the physics behind the processes occurring in different metallurgical devices. With this, it is possible to design and develop new technological solutions that improve the quality of steel products and minimize the negative impact on the environment. However, the usage of numerical approaches without proper validation can lead to misleading results and conclusions. Therefore, in this paper, the authors focus on the development of the CFD-based (computational fluid dynamics) approach to investigate the liquid steel flow inside one metallurgical device, namely a ladle furnace combined with an EMS (electromagnetic stirring) system. First, a numerical simulation of electromagnetic stirring in a scaled mercury model of a ladle furnace was carried out. The numerical results, such as stirring speed and turbulent kinetic energy, were compared with measurements in the mercury model. It was found that the results of the transient multiphase CFD model achieve good agreement with the measurements, but a free surface should be included in the CFD model to simulate the instability of the flow pattern in the mercury model. Based on the developed model, a full-scale industrial ladle furnace with electromagnetic stirring was also simulated and presented. This research confirms that such a coupled model can be used to design new types of EMS devices that improve molten steel flow in metallurgical equipment.

## 1. Introduction

The steelmaking industry has undergone significant changes in recent years to meet goals related to green steel technology and reducing carbon dioxide emissions [1,2]. Moreover, engineers are focusing on improving steel’s cleanliness and quality to satisfy increasing customer needs. To fulfill the mentioned requirements, a better understanding of the processes occurring during steel production in different vessels is needed to allow for their precise control. Such specific knowledge enables improvement of the processes controlling liquid steel behavior already at the initial stages of the production line, leading to a direct reduction in pollutant emissions to the atmosphere.

One such manufacturing stage, which is addressed in the current paper, is based on the ladle furnace. The ladle furnace is responsible for reductions in nonmetallic inclusions to improve the quality and cleanliness of steel. This improvement can be realized by controlling the flow inside the ladle furnace to increase the mixing process efficiency, which is advantageous in the case of homogenization of the steel structure and reduction of mixing time. Eventually, it ensures faster production and increases effectiveness while reducing pollutant emissions.

Different methods such as gas stirring [3], mechanical stirring [4] and electromagnetic stirring [5,6] can realize an increase in the mixing process in the ladle furnace. As EMS (electromagnetic stirring) systems seem to have great potential in increasing mixing efficiency, a proper design of a ladle furnace with EMS was tackled in the current paper.

In the past, authors [3,7] measured the effectiveness of gas stirring methods by experimental investigations. However, these kinds of methods are technically complicated, expensive and time-consuming. Moreover, such experimental investigation becomes even more demanding when a parametric study of the process is required. Therefore, the advantages provided by numerical methods are more frequently used to facilitate this investigation and support the process development. A method that is particularly suited for this class of problems is computational fluid dynamics (CFD), which enables the investigation of the flow behavior of liquids. In this case, the motion of molten steel in 3D space can be considered in the process of designing technological solutions to increase the efficiency of the ladle furnace, leading to a reduction in mixing time [8,9,10,11,12]. The method can also be used for the prediction of slag layer behavior [6,13] or more advanced simulations like desulfurization [14] and inclusion removal processes [15].

However, the main question of the current research is as follows: How accurate are such numerical simulations in the case of modeling complex mixing operations supported by EMS systems? To address this question, two variants of a complex one-way coupled model, considering electromagnetic simulation and CFD flow evaluation, were developed to match the experimental setup, allowing direct validation of the results. The investigated model variants differ in complexity and capabilities in capturing the phenomena controlling liquid steel behavior.

## 2. Coupled Model Development and Validation

**Figure 1:**
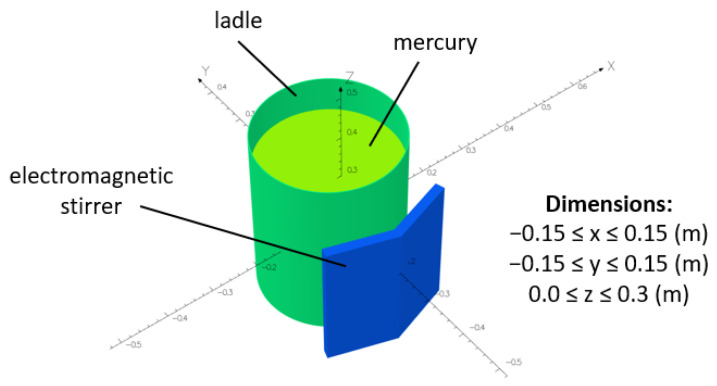
Geometry and dimensions of the numerical model used during the investigation.

For this investigation, experimental results were acquired from specifically designed lab tests based on the evaluation of mercury flow in a ladle furnace [16]. The mercury was used to allow direct visualization of the flow without the need to heat the metal to high temperatures. The research was focused on a 150-ton ladle furnace with a linear EMS system prepared on the scale of 1:10. Therefore, the ladle furnace numerical model was developed accordingly, as presented in Figure 1.

A one-way coupled electromagnetic–CFD numerical model was developed to capture interactions between the EMS system and the liquid metal.

EMS stirring is realized by the electromagnetic stirrer where the main distribution of the forces F (N/m^3^) is based on the Lorenz force calculation according to the following equation:

(1) $$F=\sigma \left(E+U\times B\right)\times B,$$

where σ—area charge density (C/m^2^), E—electric field (V/m), U—velocity (m/s) and B—magnetic field (T).

In the case of the stirring applications, the magnetic flux density and electric field applied to the molten steel are harmonic in time. The electric field can be presented as follows:

(2) $$\;E\left(t\right)=E_{0}\left(t\right)-\nabla \emptyset \left(t\right),$$

where $E_{0}\left(t\right)$—the harmonic electric field induced by the harmonic magnetic flux in the case of the stationary melt and $\emptyset \left(t\right)$—the electrostatic potential induced by the magnetic flux density and flow of the molten steel.

Based on the above, the Lorenz force can be presented as a stirring force density F (N/m^3^):

(3) $$F=\sigma \left(E_{0}-\nabla \emptyset +U\times B\right)\times B,$$

and then, after the decomposition:

(4) $$F=\sigma \left(E_{0}\times B)-\sigma \nabla \emptyset \times B+\sigma (U\times B\right)\times B,$$

The first part of (4) is not dependent on the velocity but has a major contribution to the total force density. The second part of (4) is dependent on the velocity, and this dependence can be included to obtain better accuracy of the model. To do this, the full coupling between the electromagnetic and CFD solvers can be realized to include the evolution in time. However, this solution extremely extends calculation time due to the iterative exchange of information in the fully coupled algorithm. To avoid this, in the current work, the time-averaged force was calculated for stationary molten steel, and then the additional term was added separately in the CFD solver to provide a weak coupling concept.

In this case, the harmonic force for a moving melt is averaged in time and can be presented as follows:

(5) $$\langle \vec{f}\rangle =\langle \vec{f_{0}}\rangle +\langle \vec{f_{damp}}\rangle +\langle \vec{f_{\varphi }}\rangle ,$$

(6) $$\langle \vec{f_{0}}\rangle =\frac{1}{2}Re\left\{\vec{J}\times \vec{B_{0}^{*}}\right\},$$

(7) $$\langle \vec{f_{damp}}\rangle =\frac{\sigma }{2}Re\left\{\{\vec{v}\times \vec{B_{0}}\}\times \vec{B_{0}^{*}}\right\},$$

(8) $$\langle \vec{f_{\varphi }}\rangle =\frac{\sigma }{2}Re\left\{\vec{\nabla }\varphi \times \vec{B_{0}^{*}}\right\},$$

where $\langle \vec{f_{0}}\rangle$—the time-averaged harmonic force for the melt, which is stationary, $\langle \vec{f_{damp}}\rangle$—the force damped because of the occurrence of the velocity $\vec{v}$, $\langle \vec{f_{\varphi }}\rangle$—the potential force, which is caused due to the eddy currents and magnetic field density occurrence in the moving molten steel, $\vec{J}$—current density (A/m^2^) and φ—electric potential (kg m^2^/(s^3^ A)). The harmonic dynamics for the electromagnetic fields are punctuated as bold entities, the complex conjugate is highlighted by the asterisk *, and the fields for stationary melt are depicted by the subscript 0.

Based on the above, the complex relationship can be simplified according to the literature’s assumptions [17,18] as follows:

(9) $$\langle \vec{f}\rangle =\langle \vec{f_{0}}\rangle \left(1-\frac{\langle \vec{f_{0}}\rangle \cdot \vec{v}}{\left|\langle \vec{f_{0}}\rangle \right|v_{travelling\;wave}}\right),$$

where $v_{travelling\;wave}$ is the traveling speed of the electromagnetic wave along the stirrer and can be described as follows:

(10) $$v_{travelling\;wave}=2\tau f,$$

which confirms that the factor responsible for the compensation of the forces can be presented as

(11) $$1-\frac{v}{2\tau f}.$$

This term is valid along the direction of the traveling wave. In (10) and (11), τ—pole pitch (m) and f—frequency (Hz). Both are the stirrer’s parameters.

Finally, the equation responsible for compensation of the stirring force distribution in case of the molten steel flow inside the metallurgical equipment can be presented as follows:

(12) $$\vec{F_{compensated}}=\vec{F}\left(1-\frac{\vec{F}\cdot \vec{v}}{2\tau f\cdot \left|\vec{F}\right|}\right).$$

The electromagnetic simulations were carried out with Opera 2022 software [19,20], which provides the mentioned force distribution in the whole volume of the melt. Then the interpolation technique was realized to map the forces between the two meshes to couple the results with the CFD model. Finally, Ansys Fluent 2023 R1 software incorporated these forces as additional momentum source terms. The forces were then recalculated based on the above equation to obtain the compensated force distribution. All of the operations were realized by the developed user-defined functions (UDFs).

The CFD model was developed within the framework of the finite volume method and is based on the Navier–Stokes equations specified for mass, momentum and energy conservation as follows:

Continuity equation:

(13) $$\frac{D\rho }{Dt}+\rho \frac{\partial U_{i}}{\partial x_{i}}=0,$$

Momentum equation:

(14) $$\rho \frac{\partial U_{j}}{\partial t}+\rho U_{i}\frac{\partial U_{j}}{\partial x_{i}}=-\frac{\partial P}{\partial x_{j}}-\frac{\partial \tau_{ij}}{\partial x_{i}}+\rho g_{j},$$

where

(15) $$\tau_{ij}=-\mu \left(\frac{\partial U_{j}}{\partial x_{i}}+\frac{\partial U_{i}}{\partial x_{j}}\right)+\frac{2}{3}\delta_{ij}\mu \frac{\partial U_{k}}{\partial x_{k}},$$

Energy equation:

(16) $$\rho c_{\mu }\frac{\partial T}{\partial t}+\rho c_{\mu }U_{i}\frac{\partial T}{\partial x_{i}}=-P\frac{\partial U_{i}}{\partial x_{i}}+\lambda \frac{\partial^{2}T}{\partial x_{i}^{2}}-\tau_{ij}\frac{\partial U_{j}}{\partial x_{i}},$$

where U—velocity (m/s), ρ—density (kg/m^3^), P—pressure (Pa), τ—shear stress (Pa), g—force per unit mass (m/s^2^), μ—molecular viscosity (kg/(m s)), c—specific heat (J/(kg K)), T—temperature (K) and λ—thermal conductivity (W/(m K)). Due to the fact that the experiment was conducted at room temperature, the energy equation and temperature dependencies are not taken into consideration within this work.

**Figure 2:**
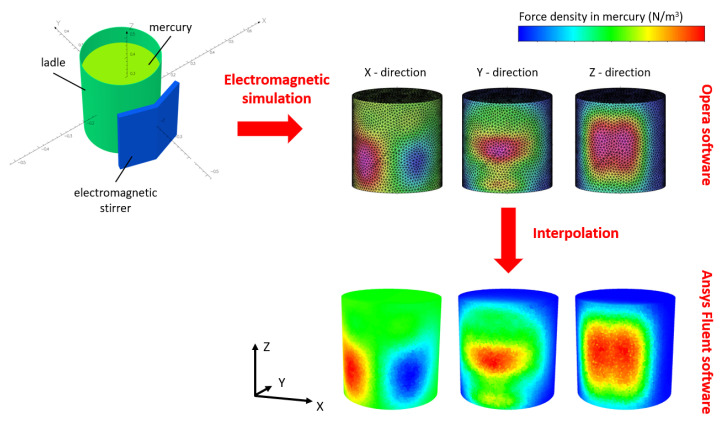
Schematic of coupling between the electromagnetic and fluid models.

The data flow in the developed one-way coupled model is summarized in Figure 2.

The developed model was adapted to replicate the mentioned experimental setup for mercury, which is liquid at room temperature. The properties of the mercury used during the research are gathered in Table 1.

During the investigation, two different classes of models were developed according to the presented procedure to evaluate their capabilities for this kind of investigation.

**Figure 3:**
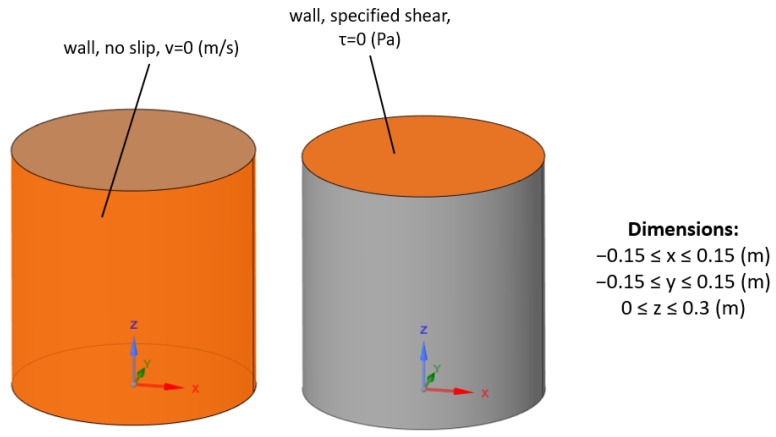
The boundary conditions applied to the liquid metal domain in the steady-state CFD simulation.

The liquid domain’s simplified steady-state model was considered first. Boundary conditions of the model were prepared according to the experimental setup and included no-slip walls in order to model the vessel’s walls and a separate virtual wall at the top with a specified shear equal to zero as a numerical free surface condition (Figure 3).

**Figure 4:**
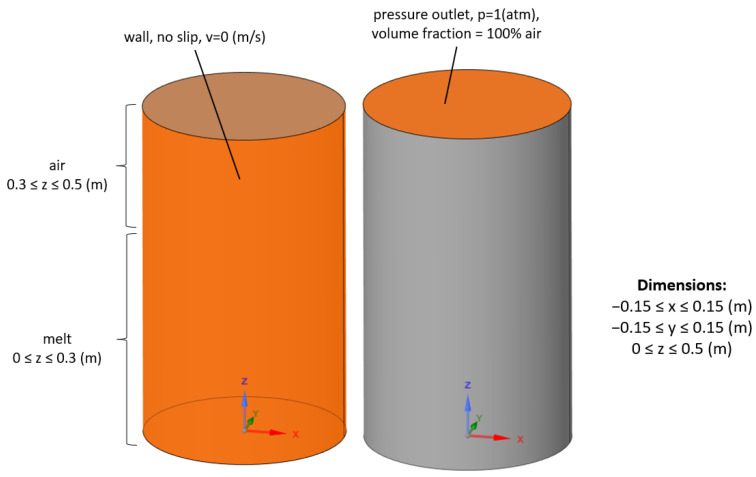
Updated boundary conditions in case of the multiphase, transient simulation.

Then, for a more detailed simulation, the transient, multiphase model was also taken into consideration. The multiphase approach is based on the VOF (volume of fluid) method [21]. In this case, the geometry of the ladle furnace was extended to include the air domain above the melt. Moreover, the boundary condition at the top wall was modified to reflect the pressure outlet with an ambient pressure of 1 atmosphere to match the pressure distribution inside the mercury. For the multiphase VOF simulations, the definition of the air volume fraction at the top was also added to properly simulate the backflow. The summary of the defined boundary conditions is presented in Figure 4.

The initialization of the multiphase model includes the assignment of the melt and air domains as described in Figure 4. Furthermore, the mixing process starts from a velocity equal to zero in the whole computational domain.

After the definition of both models, a mesh sensitivity study was conducted to evaluate the impact of the mesh density on the quality of the obtained results.

### 2.1. Mesh Sensitivity Study

**Figure 5:**
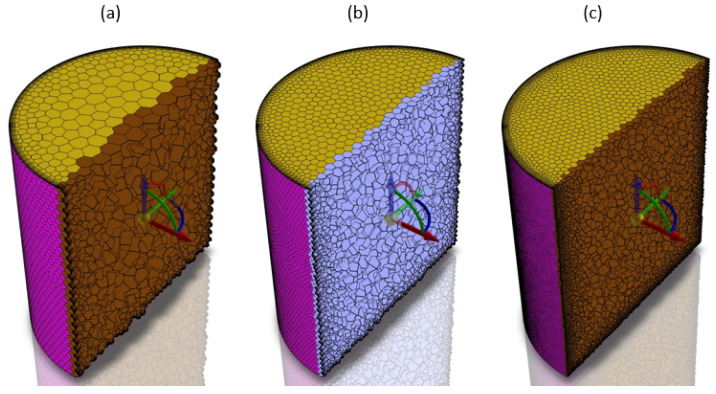
Three different mesh densities for the mesh sensitivity study with the steady-state model: (**a**) coarse, (**b**) medium and (**c**) fine.

Three different levels of mesh density were considered: coarse, medium and fine. The discretization included almost 73,000 elements in the case of the coarse mesh, over 180,000 for medium mesh and almost 750,000 for fine mesh, as seen in Figure 5.

**Figure 6:**
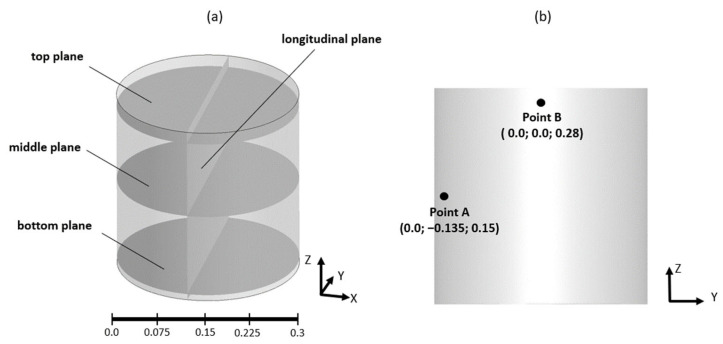
Location of the (**a**) planes and (**b**) points for qualitative and quantitative model validation and mesh sensitivity study.

**Figure 7:**
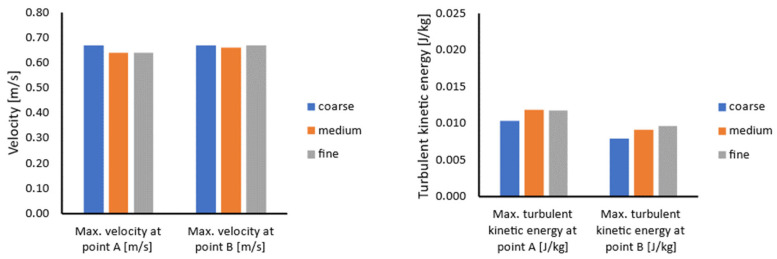
Mesh sensitivity results for the developed steady-state coupled model: maximum velocity (**left**) and maximum turbulent kinetic energy (**right**) at points.

During the experimental study, the velocity fields in different sections of the ladle furnace, as well as velocity values at selected points, were measured [16]. Therefore, the same definition of the model section planes and selected points (Figure 6) was used in the mesh sensitivity study for qualitative and quantitative data interpretation. A summary of the obtained results during the mesh sensitivity evaluation is presented in Figure 7.

The above results indicate that the values do not significantly depend on the mesh quality. The error between the fine and medium mesh results is not larger than 5%. However, the error between the medium and coarse mesh results is slightly higher at the level of 13%. Therefore, the medium mesh was used during further investigations to reduce the calculation time and maintain the quality of results.

**Figure 8:**
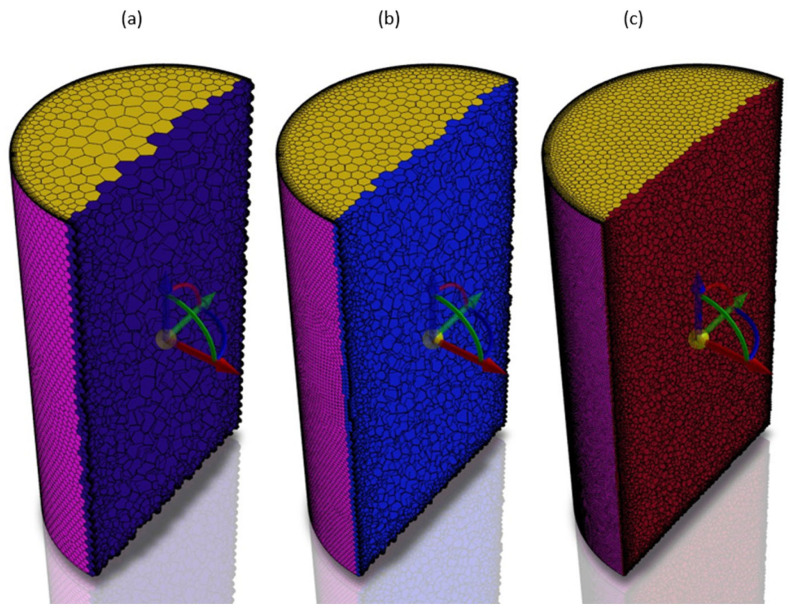
Three different mesh densities for the mesh sensitivity study with the transient model: (**a**) coarse, (**b**) medium and (**c**) fine.

**Figure 9:**
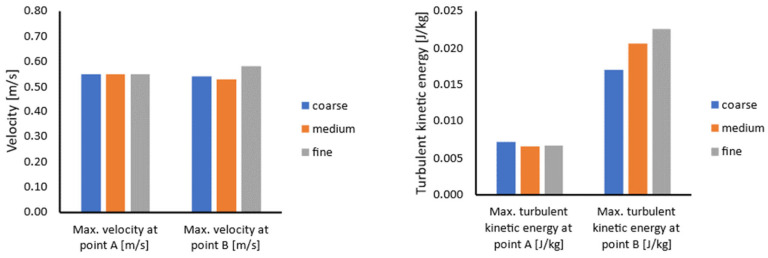
Mesh sensitivity results for the developed transient, multiphase model: maximum velocity (**left**) and maximum turbulent kinetic energy (**right**) at points.

Similarly, a mesh sensitivity study was also conducted for the transient model. In this case, again, three different mesh densities were considered: a coarse mesh with 111,000, medium with almost 387,000 and fine with above 1,000,000 polyhedral elements (Figure 8). The results of the mesh sensitivity studies are collected in Figure 9.

The transient model analysis confirmed that the fine and medium mesh difference for velocity measurements is not higher than 6%. The error between the medium and coarse meshes is more elevated, mainly in the case of turbulence kinetic energy measurements.

Based on this, again, the medium mesh was used for further calculations to avoid a long calculation time.

Finally, both models’ predictions could be compared and validated against the experimental data.

### 2.2. Validation

**Figure 10:**
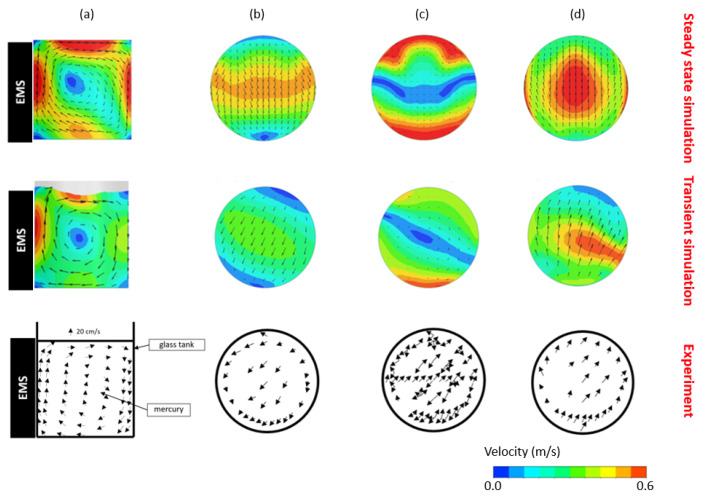
Velocity evolutions from steady-state simulation (**top**), transient simulation (**middle**) and experiment (**bottom**) for (**a**) longitudinal, (**b**) bottom, (**c**) middle and (**d**) top cross-sections.

A comparison of the developed models’ predictive capabilities with respect to the experimental measurements was carried out along the mentioned planes and direct measurements at selected points. The velocity field distribution with vectors from steady-state, transient and experimental measurements is gathered in Figure 10.

The above comparison between the simulation results and experiment suggests that the general velocity distribution in different parts of the furnace is compatible, but in the case of the steady-state CFD simulation, the rotation of the flow presented in the real case is not observed. The rotation of the flow can be caused by the following:

- Unstable behavior of turbulences, which is a random phenomenon.
- Unstable behavior of the free surface in time, which significantly influences the behavior of the flow inside the ladle and distribution of the velocities.
- The real model is not ideally symmetrical; moreover, the stirring forces are harmonic and transient.

**Figure 11:**
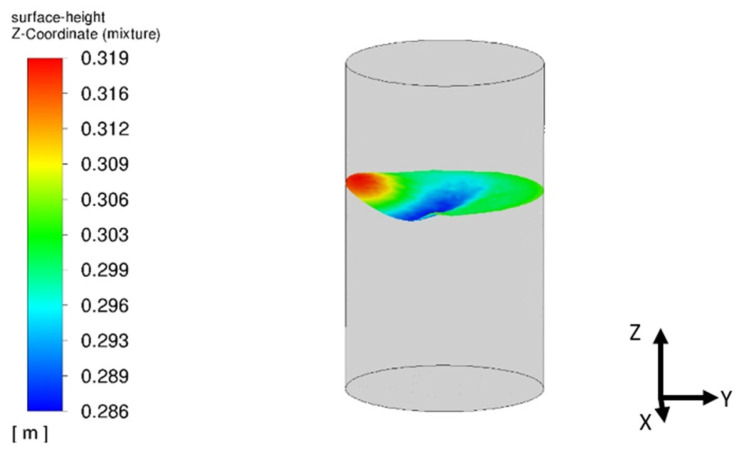
Free surface shape obtained in CFD simulation thanks to the VOF approach after 100 (s).

The mentioned free surface simulated by the multiphase model has a significant impact on the velocity distribution inside the mercury, which is well visible in the 3D view in Figure 11.

It can be summarized that the transient, multiphase simulation with the VOF approach enables us to obtain simulation results closely replicating the experimental observations. The velocity distribution had the same character as observed during the test. The experimentally visible additional rotation of the flow distribution was also observed in the numerical simulation. The laboratory measurements confirm that the asymmetric flow was present in the entire melt volume. A couple of the first circulations of mercury seem to be stable and symmetric, and after some time, the distribution turns around 20 degrees in one or another direction [16].

The direct qualitative comparison between the numerical and experimental results in the selected points in the melt from Figure 6 is presented in Table 2.

**Figure 12:**
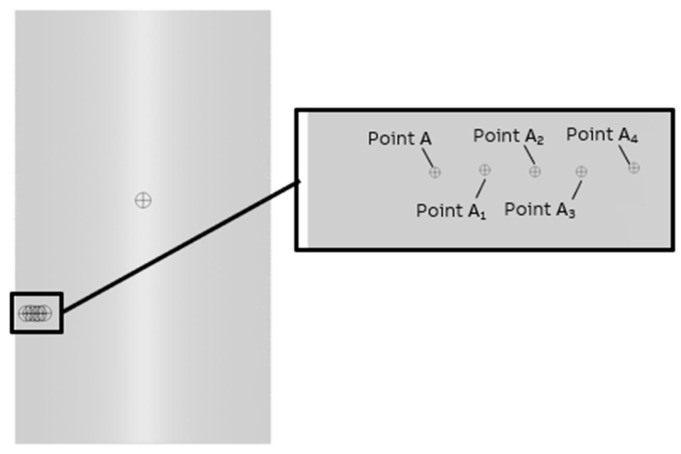
Location of new measurement points in the transient model. Distance between additional points is equal to 0.005 (m).

As seen, the transient, multiphase simulation more closely predicts the reality. The largest difference is observed in the case of the velocity and maximum turbulent energy parameter at point A (along the stirrer). It should be mentioned that the location of this point was not fully specified in the experimental measurements. The only available information suggests that the point is along the stirrer, but information about the distance from the wall of the vessel was not provided in [16]. Moreover, the numerical model includes the RANS approach, which can affect the turbulent kinetic energy parameter, and a precise selection of the measurement point is crucial in that case. Due to this, the region of interest was extended in the evaluation of the numerical results and information from four additional points was extracted, as presented in Figure 12. A summary of the maximum turbulent energy values at these points is gathered in Table 3.

The presented comparison confirms that the transient multiphase simulation results agree with the experimental findings. Therefore, this model should be used in practical computer-aided technology design of EMS systems for the ladle furnace. The steady-state approach can be used for the initial evaluation of the new ideas, but a more detailed analysis should be based on advanced multiphase modeling despite the longer simulation times.

Therefore, a full-scale study of the role of EMS in the industrial ladle furnace based on the developed and validated model can now be presented in the following part of this paper.

## 3. Evaluation of the EMS System’s Role in the Industrial Ladle Furnace Setup

**Figure 13:**
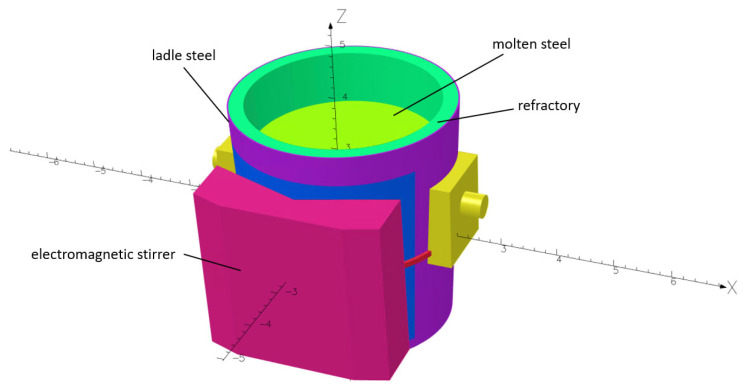
The geometry of the industrial 160-ton ladle furnace.

As mentioned, the main goal of the EMS system is to increase the mixing phenomena inside the molten steel and also control the velocity distribution thanks to the changes in the EMS parameter setup. To evaluate the influence of the EMS system on the efficiency of the liquid steel stirring operation, a 160-ton ladle furnace with a bath height equal to 2.85 (m) and with the vertical stirrer ORT1215 was selected as a case study. The geometry of the ladle furnace with the EMS stirrer is presented in Figure 13. The commercial ORT1215 EMS stirrer parameters are presented in Table 4.

As indicated above, the analysis is based on the developed coupled transient VOF model and includes the full, turbulent, incompressible flow, where the gravitational acceleration is included to model the hydrostatic pressure. The properties of the molten steel used in the model are presented in Table 5. The polyhedral mesh with a medium-type mesh density was generated in Ansys Fluent Meshing 2023 R1 software. Moreover, the geometry was additionally divided into subregions with 100 (mm) thickness, located at the bottom, middle and top parts of the molten steel to measure the values of velocity in these regions.

**Figure 14:**
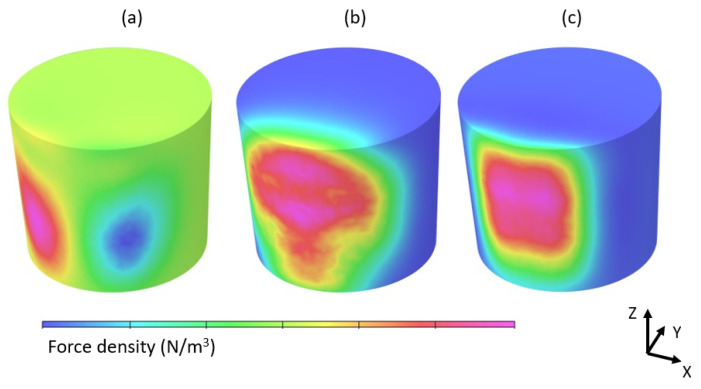
Distribution of the force density generated by the electromagnetic stirrer in (**a**) x, (**b**) y and (**c**) z directions.

Figure 14 presents the simulated force density generated by the EMS system and mapped into the CFD.

**Figure 15:**
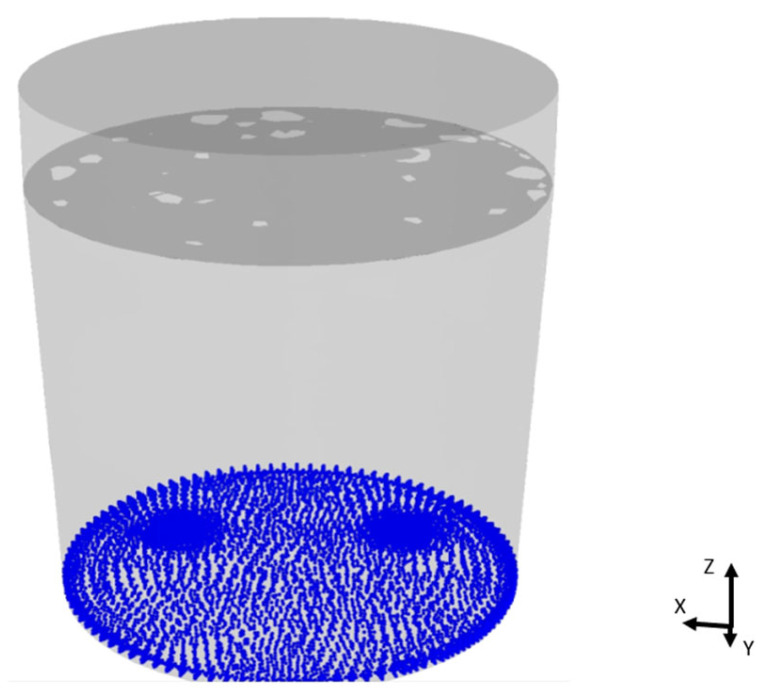
The initial location of the DPM particles used for visualization of the mixing process.

The additional massless discrete phase model (DPM) particles were included in the CFD simulation to visualize the mixing inside the furnace. DPM particles are treated only as sensors which are moving together with the molten steel and do not affect the flow in any case. Their initial location in the model is presented in Figure 15.

**Figure 16:**
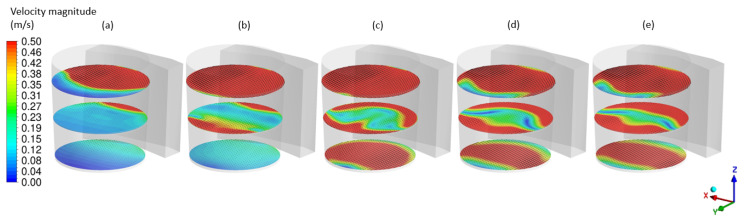
Velocity distribution on surface 100 (mm) offset from the bottom, in the middle of the melt and 100 (mm) offset from the bath surface for (**a**) 5 (s), (**b**) 10 (s), (**c**) 20 (s), (**d**) 50 (s) and (**e**) 200 (s).

**Figure 17:**
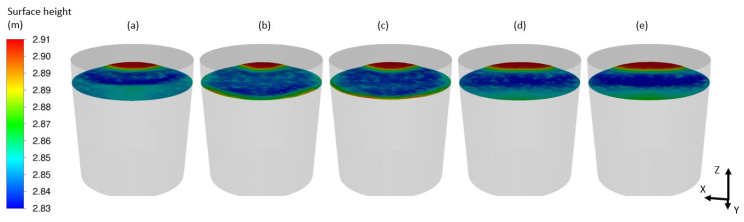
Free surface heights for (**a**) 5 (s), (**b**) 10 (s), (**c**) 20 (s), (**d**) 50 (s) and (**e**) 200 (s).

**Figure 18:**
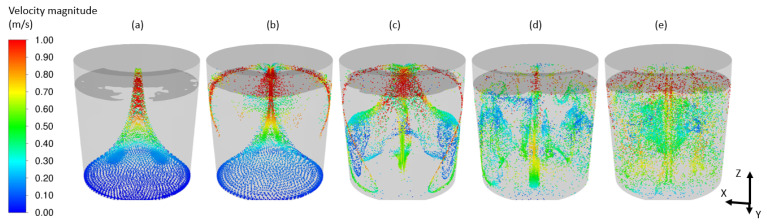
DPM massless particles representing the mixing process for (**a**) 5 (s), (**b**) 10 (s), (**c**) 20 (s), (**d**) 50 (s) and (**e**) 200 (s).

Examples of obtained results in the form of the velocity distribution (Figure 16), free surface behavior (Figure 17) and mixing process (Figure 18) are discussed below.

**Figure 19:**
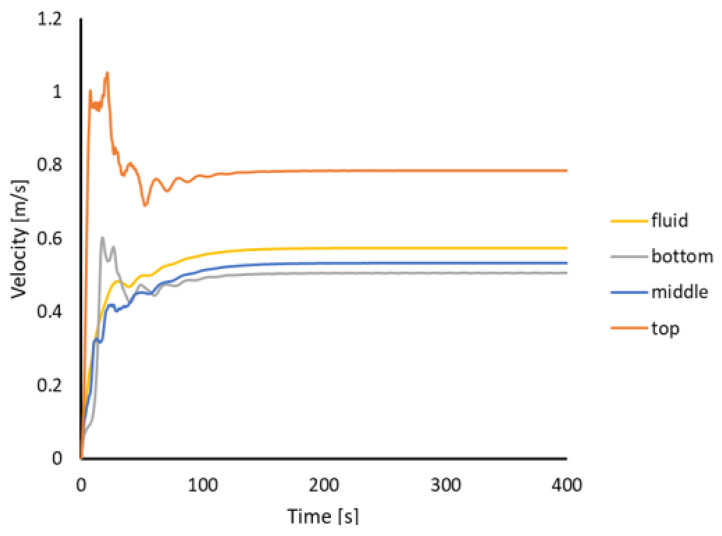
Volume-averaged velocity across the molten steel and in the bottom, middle and top parts of the ladle furnace.

Moreover, to control the velocity in different parts of the furnace, the volume-averaged speed was measured for the entire fluid and in different parts of the ladle furnace—bottom, middle and top—and the results are gathered in Figure 19.

## 4. Discussion

The presented approach of the coupling between the electromagnetic and CFD simulations confirms that numerical methods with high agreement can predict electromagnetic stirring. In this approach, the electromagnetic field for a stationary melt and also its dependence on the motion of the melt can be included. With this, direct similarity of the numerical approach to the real measurements is obtained. Even one-way coupling, which does not require high computational costs, provides comparable results. This approach can be successfully used in the standard industrial applications of the furnaces in the metallurgy industry.

More detailed analysis suggests that both the steady-state and transient approaches provide correct results, but it is worth highlighting that the steady-state numerical simulation does not give full agreement in the velocity field. The difference is caused by the simplifications with the modeling of the free surface, which is simulated by the boundary condition of the wall without shear stress. This method does not include the behavior of the free surface shape and its influence on the velocity field. The mentioned dependence is included in a more advanced, multiphase approach, which enables us to track the behavior of the free surface directly. Oscillations of the free surface cause an impact on the velocity field. Moreover, the flow inside the ladle furnace under EMS stirring is turbulent, where turbulences are random phenomena that directly influence the behavior of the flow. Moreover, the approach to modeling the EMS force is averaged in time; in the real case, the EMS stirring also generates oscillations, which can affect flow. It is worth mentioning that the obtained results are sensitive to the definition of the measurement points, and due to the lack of detailed information about the position of point A in the real experiment, an additional analysis of the dependence between turbulent kinetic energy and velocity for that point (Figure 12) was conducted to depict the importance of the input, which must be provided for a proper comparison of the results. Nevertheless, the presented approach confirms that the agreement between the multiphase, transient simulation and the real experiment is very high and can be used in further detailed analyses. The steady-state approach can be treated as an initial stage to evaluate the ranges of the velocity and turbulent kinetic energy in the ladle furnace and select the EMS model, which will be used in further analyses.

Finally, from the industrial case study it was noticed that the free surface is very stable in time and does not disturb the velocity field. This is the main reason that the velocity distribution in the whole melt is more stable, and this example can be simplified to the steady-state approach. The velocity measurements presented in Figure 19 confirm the stable character of the flow. After 200 (s), the value of the velocity in particular parts of the furnace and the whole furnace does not change in time.

The main outcome from the presented validation is that the approach can be used to evaluate the EMS mixing phenomena in real applications in the metallurgy industry, which reduces the costs and time of conduction of real experiments. Thanks to the numerical simulations, the online measurements can be omitted in the initial stage of the designing of the dedicated solutions. CFD methods provide information about the velocity distribution inside the metallurgical device, enable evaluation of the turbulent kinetic energy to understand the turbulence phenomena and also track the mixing process. Based on the initial results, the stirrer and process parameters can be controlled. The parametric study, which the numerical simulations can easily conduct, provides the solutions with the optimum setup of the stirrer, which hence prevents the wasting of energy and reduces the mixing time in real applications.

## 5. Conclusions

Based on the above-presented results, the following conclusions can be drawn:

1. One-way coupling between the electromagnetic and CFD solvers is able to obtain good agreement between the simulation and experimental results.
2. The characteristic rotation of the flow presented in the mercury is observed in the case of the VOF multiphase approach, which suggests that the approach is able to predict phenomena inside the metallurgy industry.
3. Free surface oscillations have a significant impact on the flow behavior inside the metallurgical devices and should not be omitted.
4. The steady-state approach is a simplified approach which does not reflect all of the dependencies inside the melt flow and can be treated only as an initial evaluation of the process and EMS parameters.
5. The presented approach can be successfully used in industrial applications to reduce the costs and time needed for real experiments in the initial stage of the designing of particular EMS solutions.
6. Thanks to the numerical methods, the velocity and turbulent kinetic energy can be measured and tracked during the solution, which enables controlling their ranges and changing the EMS stirrer parameters if needed. Moreover, the mixing process can be observed to understand the character of the flow to prevent the occurrence of dead zones. The numerical approach enables us to understand the free surface behavior and its influence on the flow.

## Figures and Tables

**Table 1:** Mercury properties at room temperature.

Mercury at 20 (°C)	
Density (kg/m^3^)	13,545
Viscosity (kg/(m s))	0.00154413

**Table 2:** Comparison between the experimental, steady-state and transient VOF results.

Method	Experiment	Steady-State Simulation	Transient Simulation
Max. speed at point A (m/s)	0.40	0.64	0.55
Max. speed at point B (m/s)	0.45	0.66	0.53
Max. turbulent kinetic energy at point A (J/kg)	0.0040	0.0119	0.0066
Max. turbulent kinetic energy at point B (J/kg)	0.0170	0.0156	0.0206

**Table 3:** Velocity and turbulent kinetic energy for different locations of point A.

Method	Point A	Point A_1_	Point A_2_	Point A_3_	Point A_4_	Experiment
Max. speed (m/s)	0.55	0.50	0.49	0.45	0.42	0.45
Max. turbulent kinetic energy (J/kg)	0.0066	0.0072	0.0074	0.0076	0.0077	0.0040

**Table 4:** EMS system process parameters.

ORT1215	
Active power (kW)	338.7
Current (A)	1350
Frequency (Hz)	1.1
Pole pitch (m)	1.46

**Table 5:** Steel properties of steel used during the CFD simulation.

Steel Properties	
Density (kg/m^3^)	6900
Viscosity (kg/(m s))	0.069

## Data Availability

The raw/processed data required to reproduce these findings cannot be shared at this time as the data form part of an ongoing study.
